# A Location Selection Policy of Live Virtual Machine Migration for Power Saving and Load Balancing

**DOI:** 10.1155/2013/492615

**Published:** 2013-11-17

**Authors:** Jia Zhao, Yan Ding, Gaochao Xu, Liang Hu, Yushuang Dong, Xiaodong Fu

**Affiliations:** ^1^College of Computer Science and Technology, Jilin University, Changchun, Jilin 130000, China; ^2^Key Laboratory of Symbolic Computation and Knowledge Engineering of Ministry of Education, Jilin University, Changchun, Jilin 130000, China

## Abstract

Green cloud data center has become a research hotspot of virtualized cloud computing architecture. And load balancing has also been one of the most important goals in cloud data centers. Since live virtual machine (VM) migration technology is widely used and studied in cloud computing, we have focused on location selection (migration policy) of live VM migration for power saving and load balancing. We propose a novel approach MOGA-LS, which is a heuristic and self-adaptive multiobjective optimization algorithm based on the improved genetic algorithm (GA). This paper has presented the specific design and implementation of MOGA-LS such as the design of the genetic operators, fitness values, and elitism. We have introduced the Pareto dominance theory and the simulated annealing (SA) idea into MOGA-LS and have presented the specific process to get the final solution, and thus, the whole approach achieves a long-term efficient optimization for power saving and load balancing. The experimental results demonstrate that MOGA-LS evidently reduces the total incremental power consumption and better protects the performance of VM migration and achieves the balancing of system load compared with the existing research. It makes the result of live VM migration more high-effective and meaningful.

## 1. Introduction

VM technology [1, 2], one of the most important technologies in cloud computing, is not only a way to implementing cloud computing such as infrastructure as a service (IaaS) [3] architecture but also the embody of the cloud computing idea, whereas live VM migration technology, which is widely used for the maintenance management in virtualized cloud computing data centers, is the representative of the VM technologies. When a VM needs migrating from source host to target host for some goal or several goals, generally, the migration target of a VM is chosen randomly as long as the host can accommodate it, and then one can automatically or manually move the VM to a target host. It is obvious that the way to randomly choose a target host for a live VM migration, which some event has aroused and has more than one available target host to meet the requirements of that event, is not efficient in all respects. Therefore, a high-efficient location selection policy to migrate the migrant VMs onto the right fit hosts is necessary.

Nowadays, power consumption of data centers has a huge impact on environments. Researchers have been seeking to find effective solutions to minimize power consumption of data centers while keeping the desired quality of service. On the background of low-carbon world and cloud computing era, researchers have already proposed the field of green cloud computing based on cloud computing and virtualization as well as aiming at reducing power consumption in cloud computing data centers. 

There are a large number of VMs and tasks running on the hosts of cloud data centers. Some hosts have a heavy load which has a huge impact on the service performance. And some hosts have a relatively lighter load which results in a low utilization of resources. Therefore, it is important to achieve load balancing in cloud data centers as it has covered many key respects of cloud computing data centers. 

In this paper, we have focused on the live VM migration policy for power saving and load balancing. Generally speaking, the migrant VM has many available target hosts. However, only one target host is most suitable for the VM in order to achieve the management goals, which include minimizing the total incremental power consumption in cloud data center and achieving load balancing as much as possible in the case that the performance constraint of live VM migration is met.

Many papers have presented some heuristic algorithms to find optimal solutions aiming to minimize power consumption. However, most research has only taken power consumption into consideration but not considered load balancing. In other word, the current research has focused on a single objective optimization for minimizing the power consumption. In this paper, we have focused on the power consumption and load balancing. It is a multiobjective optimization problem (MOP) which differs from the traditional single objective optimization problem. The basic idea of the existing research is that, according to the current situation and history of a cloud data center, the controller has searched for a best migration policy by optimizing the objective function of the proposed algorithm. In the MOPs, the situation is different and complex. It needs to optimize multiple objectives at the same moment. Generally, these objectives influence each other and are even contradictory. To achieve a multiobjective optimization, the problem of how to evaluate and compare two solutions for the multiple functions as well as the problem of how to give each solution a reasonable fitness value towards multiple functions must be addressed first. 

It is well known that it is the Pareto dominance principle that is used for MOPs to address the problems. According to the Pareto theory, we know that the optimal solution of an MOP is not a single solution but a set of Pareto optimal solutions. The common and classical heuristic algorithms are used to convert the MOP to a single-objective optimization problem by emphasizing one particular Pareto optimal solution at a time. When such an algorithm will be used to search out multiple solutions, it has to be executed many times, hopefully finding a different solution at each simulation run. However, the genetic algorithm (GA), as the representative of evolution algorithms (EAs), is suitable for dealing with the MOPs since it has the ability to find multiple Pareto optimal solutions in one single simulation run. As EAs work with a population of solutions, a simple EA can be extended to maintain a diverse set of solutions. With an emphasis for moving towards the true Pareto optimal region, an EA can be used to find multiple Pareto optimal solutions in one single simulation run [4, 5]. In this paper, we have proposed a novel heuristic GA-based location selection policy MOGA-LS of VM for power saving and load balancing, which utilizes the Pareto nondominated sorting, individual density estimation, and mathematical statistics of Pareto optimal solutions to achieve a long-term excellent power saving and load balancing optimization. The final experimental results have demonstrated that the MOGA-LS approach not only reduces the total incremental power consumption but also achieves a better load balancing while relatively maximizing the performance of live VM migration. That is, it has reduced the failure events of live VM migration and contributed to achieving better green cloud data centers with load balancing.

The rest of the paper is organized as follows. In Section 2, we present the related work, and the reasonable prerequisites are shown clearly. In Section 3, the main idea, design, problem formulation, solution representation, and implementation of MOGA-LS are introduced in details. In Section 4, the experimental results and analysis on CloudSim platform are given. Finally, in Section 5, we summarize the full paper, and future work is put forward.

## 2. Related Work

As far as we know, the proposed problem which refers to finding a fit target host for a live VM migration according to the standard of minimizing the increment power consumption or load balancing has not been widely researched in the related fields of live VM migration policy let alone both. However, most researchers have focused on some problems which are similar to the proposed problem in this paper. Thus, the related work of the kind of problems relating to live VM migration towards power saving and load balancing will be discussed briefly in this section.

Rusu et al. in [6] have presented a cluster-wide QoS-aware technique that dynamically reconfigures the cluster to reduce energy consumption during periods of reduced load. The proposed system consists of two important components, namely, front end manager and a local manager. While the front end manager finds the servers which should be turned on or off in terms of a given system load, the local manager will utilize dynamic voltage and frequency scaling (DVFS) technique to conserve energy. The main shortage of the approach is the on/off policy. It relies on the table of values and needs offline computing. However, the system does not make use of server consolidation through VM migration, and thus, its on/off policy may not be much effective.

Srikantaiah et al. [7] have investigated the problem of dynamic consolidation of applications serving small stateless requests in data centers to minimize the energy consumption. They modeled the problem as a multidimensional bin packing problem. However, the proposed model doesn't describe the degradation of performance due to the consolidation. Besides, the energy drawn may rely on a particular set of applications combined on a computer node. A heuristic for the defined bin packing problem is proposed by the authors. The heuristic is based upon the idea of minimizing the sum of the current allocations' Euclidean distances to the optimal point at each server. The application workload will be allocated to a server using the proposed heuristic since a request to execute a new application is received. Without the sufficient capacity of all active servers, the system will switch on a new server while reallocating all the applications using the same heuristic in an arbitrary order. The proposed approach is fit for heterogeneous environments; however, it has several shortcomings. First, the approach assumes that all applications' resource requirements are known in advance and constant. Second, performance and energy overhead, which the authors do not take account into, is caused by migration of state-full applications between nodes. The frequent switching servers on/off also generates significant costs which are not negligible for a real-world system.

Verma et al. [8] have contributed energy and migration cost-aware application placement by exploiting the energy management capabilities of virtualization. The authors have designed a new application (virtual machines) placement architecture called pMapper. It consists of three major parts, namely, a performance manager to dynamically resize the VM, an energy manager for CPU throttling, and a migration manager to identify the target host for migration using a knowledge base. They have expounded that for energy-aware scheduling approaches, estimates of energy values are not required, and only if the scheduling algorithm has abilities in finding out which server minimizes the incremental increase in total energy owing to the new VM being placed, it can place the given VM to an appropriate host. In pMapper, two algorithms are implemented. One is First Fit Decreasing (FFD) by which more energy-efficient servers are utilized first without balancing the load. The other is incremental First Fit Decreasing (iFFD) which considers the fixed target utilization of each server and achieves server consolidation by live VM migration. The proposed pMapper architecture minimizes energy and migration costs while ensuring the performance. Our approach is based on a heuristic approach which exploits the concept of minimizing total increase in the incremental energy due to the new VM migrations. The proposed architecture is simple and does not need any knowledge base to achieve significant reduction in the energy consumption.

Li et al. in [9] have proposed an approach named EnaCloud, which enables application of live placement dynamically with consideration of energy efficiency in a cloud platform. In EnaCloud, they use a virtual machine to encapsulate the application, which supports applications' scheduling and live migration to minimize the number of running machines, so as to save energy. In particular, the application placement is abstracted as a bin packing problem, and an energy-aware heuristic algorithm is proposed to get an appropriate solution. In addition, an overprovision approach is presented to deal with the varying resource demands of applications. However, the overprovision approach has risk in optimizing this problem. It may cause more cost in order to reduce cost with a certain probability.

Jeyarani et al. [10] have proposed self-adaptive particle swarm optimization (SAPSO) for efficient virtual machine provisioning in cloud aimed at that when mapping a set of VM instances onto a set of servers from a dynamic resource pool, the total incremental energy drawn upon the mapping is minimal and does not compromise the performance objectives. The advantage of the proposed solution is obvious. It has focused on not only improving the performance of workload facilitating the cloud consumers but also developing the energy efficient data center management to facilitate cloud providers. However, the approach still may be inefficient and may cause some additional events and costs from a long-term perspective as it does not take the future workload into account. Our proposed algorithm MOGA-LS is a heuristic approach which is based on PSO, one of swarm intelligence algorithms and introduces the simulated annealing (SA) idea into it.

Jing and She [11] have proposed a novel model for this problem of live VM migration for load balancing based on fuzzy TOPSIS to detect the hotspots and balance load. The proposed model is to migrate VMs between hosts using fuzzy TOPSIS theory to make decision over the whole of active hosts of the data center and detect the hotspots. The proposed model can be a suitable tool to rank hosts. However, the kind of load balancing policies based on sorting hosts is not heuristic enough and has a relatively complex computation. It is not efficient for achieving load balancing.

Yang et al. in [12] have proposed a load balancing algorithm based on live VM migration. The proposed algorithm includes two major policies: trigger strategy based on the load prediction and selection strategy of the destination node based on the multiple criteria decision. Yet the kind of load balancing policies based on load predicting is not accurate enough and needs to maintain additional historical data, as results in the unnecessary system load.

In this paper, MOGA-LS has a prerequisite. We know that MOGA-LS is to find the target host of each VM from all *m* hosts for the *n* migrant VMs. We assume that each VM's target host found by MOGA-LS will not be the host which the VM is moved out from. The algorithm provides service to live VM migration aiming at green cloud data center with load balancing. Thus, the fact that the VM should be moved out for some reason is the premise of our approach. Objectively, the prerequisite is justified from a certain perspective. Since a VM needs to be migrated from its source host, its candidate hosts will not include its source host. Otherwise, it does not need a migration event. It can be seen that for all the migrant VMs, the hosts each of which is the source host of some migrant VM will not be the target hosts. Therefore, the proposed prerequisite is reasonable and does not impact the performance and efficiency of our approach. 

## 3. Proposed MOGA-LS Approach

### 3.1. Main Idea

The proposed MOGA-LS approach is a heuristic live VM migration policy, which is used for the target location selection of live VM migration. It is a multiobjective optimization algorithm which is based on the improved GA that utilizes Pareto theory to achieve the selection, crossover, and mutation operators of GA towards multiple objectives and thus to find out the set of Pareto optimal solutions of the proposed multiobjective problem by the evolution of the population. After obtaining the set of Pareto optimal solutions, in order to get the final migration policy, we do not randomly select a solution but utilize the mathematical statistics theory to obtain the final solution of location selection of live VM migration by using a probability wheel for each VM. 

Specifically, we have employed a novel policy which is used to generate the initial population and defined a Pareto constraint dominance relation towards the proposed constrained problem to compare two solutions in the improved GA-based approach. The nondominated sorting policy and density estimation policy used for the population has been presented to make each individual of the population obtain fitness values in each generation. We have employed the tournament selection operator for the selection operator of GA. For the crossover and mutation operators, we have designed the arithmetic crossover operator and the dynamic nonuniform mutation operator. Besides, the (*μ* + *λ*) selection policy, which makes elitism that helps in achieving better convergence be introduced into the proposed MOGA-LS approach, is employed to generate the next population.

### 3.2. Design of the MOGA-LS Approach

The MOGA-LS approach is an algorithm based on multiobjective GA achieving a live VM migration policy for minimizing the incremental power consumption caused by migrating these migrant VMs onto their target hosts and making the load of cloud data center balanced after migrating under the constraint of maximizing the performance that the number of success of live VM migration events is maximized. To achieve a multiobjective GA, we have introduced the concept of Pareto optimal solutions into GA and designed a constrained Pareto dominance method to evaluate the individuals of population and assign fitness values to them as well as designing GA's genetic operators including selection, crossover, and mutation operators as well as the policy of generating the new population.

#### 3.2.1. Problem Formulation

We now formulate the proposed problem of migrating *n* VMs onto *m* hosts. Its solution can be represented by an *n* dimension of vector, each element of which denotes the target host of the migrant VM which its location represents. We assume that there are *m* available hosts in the resource pool of a cloud data center and the hosts are heterogeneous and dynamic while using space shared allocation policy. The hosts change their state dynamically according to the load. Similarly, each VM is associated with the required computing resource. It is considered that the VMs are independent of each other and are equal in priority. 

The problem can be stated as follows. Find a VM-host mapping set LS of location selections such that the total incremental power consumption caused by the migrated VMs onto hosts is minimized and after migrating the load is balanced as much as possible, while maximizing the performance by fulfilling the resource requirements of maximum number of VMs. We define a quintuple *S* = {PH, VM, PC, RL, LS} for our problem scenario. PH is a set of *m* available physical hosts denoted by PH (*m*, *t*) = {PH1, PH2, PH3 …, PH*m*}, available at migrating start time *t*. VM is a set of *n* migrant VMs denoted by VM (*n*, *t*, Δ*t*) = {VM1, VM2, VM3, …, VM*n*} accumulated within a time window Δ*t*. PC (*m*, *t*) = {*P*1, *P*2, …, *Pm*} is the power consumption by the *m* physical hosts in the resource pool. RL (*m*, *t*) = {{*R*_CPU_1, *R*_MEM_1}, {*R*_CPU_2, *R*_MEM_2}, {*R*_CPU_3, *R*_MEM_3}, …, {*R*_CPU_ *m*, *R*_MEM_ *m*}} represents the residual CPU and memory resource of each host at migrating start time *t*. The problem is multimodal, generally having more than one location selection which meets the performance constraint of the VM requests. Our goal is to find a location selection which meets the performance constraint while minimizing the incremental power consumption and minimizing the standard deviation of the residual load rates of all available hosts to achieve load balancing. That is, the goal is to obtain all Pareto optimal solutions. It can be seen as a constrained multiobjective Pareto optimization problem. 

To fulfill the performance constraint, a metric denoted by *η*_*r*_ representing resource fulfillment requirement, *η*_*r*_ represents the number of success of live VM migration events and should be equal to *n* as a constraint function ideally, is defined as follows:

(1) ηr=∑i=1n∑j=1mtij=n, i∈{1,2,3,…,n},  j∈{1,2,3,…,m}, r∈{1,2,3,…,s},

where *τ*_*i*_^*j*^ denotes the location selection of *i*th VM on the *j*th host and is defined as follows, *s* denotes the total number of location selections:

(2) $$\begin{array}{c} \tau_{i}^{j}=\{\begin{array}{cc} 1 & \\ \text{if}\;{\text{VM}}_{i}\;\text{has}\;\text{been}\;\text{allocated}\;\text{to} & \\ {\text{Host}}_{j}\;\text{and}\;{\text{rcrVM}}_{i}\leq {\text{acrHost}}_{j}, & \\ i\in \left\{1,2,3,\ldots ,n\right\} & \\ 0 & \\ \text{if}\;\text{V}{\text{M}}_{i}\;\text{has}\;\text{been}\;\text{allocated}\;\text{to} & \\ {\text{Host}}_{j}\;\text{and}\;{\text{rcrVM}}_{i}\leq {\text{acrHost}}_{j}, & \\ j\in \left\{1,2,3,\ldots ,m\right\} & \\ \text{Invalid} & \\ \text{if}\;\text{V}{\text{M}}_{i}\;\text{has}\;\text{been}\;\text{allocated}\;\text{to}\;{\text{Host}}_{j}. & \end{array} \end{array}$$

In (2), rcrVM_*i*_ denotes the minimum computing resource requirements of *i*th VM and acrHost_*j*_ denotes the available computing resource of *j*th host.

The first objective function is based on power consumption. The migration of successive VMs is represented as LS [*m*, *n*, *r*, *t* + *t*_0_(*k*)] where *r* represents any one of *s* location selections and *k* is an integer increasing with successive migrating, representing a stage. We can understand that if *k* is 3, the approach will migrate the third VM to the host which is denoted at the third location of the *r* vector. The *s* location selections at stage *k* are represented as LS [*m*, *n*, *r*, *t* + *t*_0_(*k*)]  *r* ∈ {1,2, 3,…, *s*} and their corresponding power consumption is represented as *ξ*_*k*_^*r*^. Its meaning is that according to *r* location vector after the system has migrated the *k*th VM to its target host, the total power consumption by the cloud data center is *ξ*_*k*_^*r*^, ideally. The meaning of *ξ*_*k*−1_^*r*^ can be imaged. In this paper, these parameters of *ξ*_*k*_^*r*^ can be obtained by using simulation platform in the experiment. Now, the incremental power consumption due to migrating LS  [*m*, *n*, *r*, *t* + *t*_0_(*k*)] with respect to previous migration stage LS  [*m*, *n*, *r*, *t* + *t*_0_(*k* − 1)] is defined by

(3) $$\begin{array}{c} \Delta P=\left(\xi_{k}^{r}\right)-\left(\xi_{k-1}^{r}\right);\;r\in \left\{1,2,3,\ldots ,s\right\}. \end{array}$$

For better power saving, the following *δP* ^*r*^ should be minimized, and it is denoted as follows:

(4) $$\begin{array}{c} \delta P^{r}=\sum_{k=1}^{n}\left(\left(\xi_{k}^{r}\right)-\left(\xi_{k-1}^{r}\right)\right);\;r\in \left\{1,2,3,\ldots ,s\right\}. \end{array}$$

Therefore, the proposed MOGA-LS approach minimizes *δP* ^*r*^ for power efficiency. Function (4) is the first objective for MOGA-LS.

The second objective function is based on load balancing. In this paper, we have presented the residual load rate to measure the load situation of each host. The calculation method of the residual load rate is described as follows. Now, assume that the set of available hosts is PH (*m*, *t*) = {PH1, PH2, PH3 …, PH*m*} after a time window Δ*t*. Within the Δ*t*, the set of the accumulated live VM migration requests is represented as VM (*n*, *t*, Δ*t*) = {VM1, VM2, VM3,…, VM*n*}. After migrating all the migrant VMs based on a location selection *r* ∈ {1,2, 3,4, 5,6,…, *s*}, the residual load *R* ^*ir*^ of the host *i* is defined as follows:

(5) Rir=αRCPUir+βRMEMir, i∈{1,2,3,…,m},r∈{1,2,3,…,s},α+β=1,

where *R*_CPU_^*ir*^ and *R*_MEM_^*ir*^ represent the residual CPU and memory resource of host *i* after migrating according to the solution vector *r*. So, the residual load rate *E* ^*ir*^ of host *i* is defined as follows:

(6) $$\begin{array}{c} E_{}^{ir}=\frac{R^{ir}}{T^{i}},\;i\in \left\{1,2,3,\ldots ,m\right\},\;\;r\in \left\{1,2,3,\ldots ,s\right\}, \end{array}$$

where *T* ^*i*^ can be represented as follows:

(7) $$\begin{array}{c} T^{i}=\alpha T_{\text{CPU}}^{i}+\beta T_{\text{MEM}}^{i},\;i\in \left\{1,2,3,\ldots ,m\right\}, \end{array}$$

where *T*_CPU_^*i*^ denotes the total CPU resource of host *i* and *T*_MEM_^*i*^ denotes the total memory resource of host *i*. In this paper, we think that, in order to make the load of all *m* physical hosts balanced as much as possible, the residual load rate *E* ^*ir*^ of each host should be as similar as possible after having migrating all migrant VMs accumulated within a time window Δ*t*. Therefore, we have utilized the standard deviation of all hosts' residual load rates to formulate this problem. The formula of the expectation and the standard deviation is as follows:

(8) $$\begin{array}{c} E\left(X\right)=\frac{\sum_{i=1}^{N}X_{i}}{N},\\ \sigma =\sqrt{\frac{1}{N}\sum_{i=1}^{N}{\left(X_{i}-E\left(X\right)\right)}^{2}}. \end{array}$$

By using the above two formulas, the second objective function can be described as follows:

(9) σr=1m∑i=1m(αRCPUir+βRMEMirαTCPUi+βTMEMi−∑k=1m((αRCPUkr+βRMEMkr)/(αTCPUk+βTMEMk))m)2.

So far, we have formulated the proposed problem as a multiobjective optimization problem with a constraint. That is,

(10) Min⁡{δPr=∑k=1n((ξkr)−(ξk−1r)),σr=1m∑i=1m(αRCPUir+βRMEMirαTCPUi+βTMEMi−∑k=1m((αRCPUkr+βRMEMkr)/(αTCPUk+βTMEMk))m)2, r∈{1,2,3,…,s}s.t.ηr=∑i=1n∑j=1mϕij=n, i∈{1,2,3,…,n},  j∈{1,2,3,…,m},  r∈{1,2,3,…,s}.

#### 3.2.2. Relevant Concepts of Pareto Optimal Solutions

In a MOP, the fitness values cannot be compared between multiple objectives. And generally these objectives are in conflict with each other. The improvement of an objective is often at the expense of impairing the fitness value of the other objective. Thus, it can be seen that the solution of a MOP is not only one but a set consisting of many solutions which cannot compare with each other. These solutions are called Pareto solutions set. In all Pareto solutions sets of a MOP, the best one is Pareto optimal solutions set. To explain this problem, we have given some definitions in the following.

*Pareto Dominance*. That a solution vector *u* = (*u*_1_, *u*_2_,…, *u*_*n*_) dominates the other solution vector *v* = (*v*_1_, *v*_2_,…, *v*_*n*_)  (*u*≺*v*) refers to that for all *i* ∈ {1, 2, 3,…, *k*}  *f*_*i*_(*u*) ⩽ *f*_*i*_(*v*) and ∃*j* ∈ {1, 2, 3,…, *k*} s.t. *f*_*j*_(*u*) < *f*_*j*_(*v*). In the definition, the MOP is a minimization problem by default.

*Pareto Optimal Solutions.* That a candidate solution *X* = (*x*_1_, *x*_2_, …, *x*_*k*_) ∈ *Ω* is a Pareto optimal solution refers to that ¬∃*X*′ ∈ *Ω* s.t. *X*′≺*X*.

*Pareto Optimal Solutions Set*. *P* = {*X* ∈ *Ω* | ¬∃*X*′ ∈ *Ω* : *X*′≺*X*}.

*Pareto Front*. PF = {*F*(*X*) = (*f*_1_(*X*), *f*_2_(*X*), …, *f*_*k*_(*X*)) | *X* ∈ *P*}.

As can be seen from these definitions, our goal is to find Pareto optimal solutions set. That is, the MOGA-LS approach should return the Pareto optimal solutions set finally. We have utilized Pareto dominance to compare any two individuals of population. As our proposed problem has a constraint, we have redefined the Pareto dominance as a constrained Pareto dominance as follows. A solution *i* is said to constrained-dominate a solution *j*, if any of the following conditions is true.

1. Solution *i* is feasible and solution*j* is not.
2. Solutions *i* and *j* are both infeasible, but solution *i* has a smaller overall constraint violation.

In the proposed problem, this scenario refers to that solution *j* results in more failure events of live VM migration than solution *i*. This means that if neither of the two solutions can make all live VM migration events accumulated within a time window Δ*t* successful, the solution which results in less failure events of live VM migration is better since that the most migrant VMs are migrated successfully is the premise of the proposed problem. After all, the MOGA-LS approach is a live VM migration policy.

(3) Solutions *i* and *j* are feasible and solution *i* dominates solution *j*.

In this paper, the Pareto dominance mentioned by the proposed MOGA-LS approach refers to the newly defined constrained Pareto dominance. 

#### 3.2.3. Solution Representation and GA Encoding

In order to design an efficient GA-based algorithm for finding the optimal vector of the target hosts of all migrant VMs in a time window Δ*t*, the primary problem is the solution representation as it represents a direct relationship between the problem domain and the individuals in GA. During the applying of a GA-based algorithm, we know that an individual is denoted as a solution of the specific problem. Here, there are *n* VMs to be migrated into *m* physical hosts. So, the proposed problem is an *n* dimensional problem, leading to that the individuals are represented as some *n* dimensional vectors. Each dimension has a discrete set of all possible location selections which are denoted as integers and limited to *m*. A solution vector called an individual or a chromosome is denoted by *x*_*i*_^*k*^ = (*x*_*i*1_^*k*^, *x*_*i*2_^*k*^,…, *x*_*ij*_^*k*^,…, *x*_*iD*_^*k*^), where the *x*_*ij*_^*k*^ value represents the coded value (the no. of target host of the *j*th migrant VM) of the *j*th gene (the *j*th migrant VM) of *i*th individual (the *i*th possible solution vector) in *k*th generation. 

#### 3.2.4. Design of Genetic Operators in MOGA-LS

(*1) Selection Operator.* In the MOGA-LS approach, we have employed the tournament selection operator. Our main idea is that the algorithm randomly chooses two groups of individuals from the population. Each group consists of *k* individuals. From the efficiency and diversity of the MOGA-LS approach to consider, the tournament scale *k* is set to 2 in this paper. That is, the algorithm randomly chooses two groups, each of which includes two individuals from the original population. The two winning individuals of the two groups are obtained by the comparison within groups. In the next step, the two individuals will be used for the crossover operator.

(*2) Crossover Operator.* As mentioned above, the GA encoding has employed the positive integer coding, and the integer is limited to *m*. We have not utilized the widely used Simulated Binary Crossover (SBX) crossover method that for a random given crossover point, the two parent individuals exchange the sections located on both sides of the crossover point. However, we have designed the nonuniform arithmetic crossover operator and introduced it into our approach in order to improve the global search ability and better keep the diversity of population. Let *X*_*i*_^*t*^ and *X*_*j*_^*t*^, respectively, represent the real encoding values of the crossover points of the two parent individuals *i* and *j* in the *t*th generation. After the crossover, the corresponding gene encoding values *X*_*i*_^*t*+1^ and *X*_*j*_^*t*+1^ of the two individuals are defined as follows:

(11) Xit+1=αXit+(1−α)Xjt,Xjt+1=(1−α)Xit+αXjt,

where *α* is a parameter and is not a constant. It is related to the evolution generation number. The specific definition of *α* in this paper will be described in the following.

(*3) Mutation Operator.* MOGA-LS is a heuristic approach based on GA. As a heuristic optimization algorithm, it should have better global search ability in the early iterations, and it should have better local search and convergence ability in the later iterations. Therefore, we have utilized the dynamic nonuniform mutation operator to make the scope of the gene mutation change with the increase of the generation number and thus to improve the search and convergence ability of MOGA-LS. Now, we assume that an individual (a chromosome) *X* = (*X*_1_, *X*_2_,…, *X*_*k*_,…, *X*_*n*_) is mutated to a new individual *X*′ = (*X*_1_, *X*_2_,…, *X*_*k*_′,…, *X*_*n*_). If the gene encoding value *X*_*k*_ of the mutation point *k* ranges within [*U*_min⁡_^*k*^, *U*_max⁡_^*k*^], the new gene *X*_*k*_′ is determined by the following formula:

(12) Xk′={Xk+(Umax⁡k−Xk)(1−γk(1−(t/T))ε),if  r  and  (0,1)∈[0,0.5),Xk−(Xk−Umin⁡k)(1−γk(1−(t/T))ε),if  r  and  (0,1)∈[0.5,1],

where *γ*_*k*_ is a random number distributed uniformly between 0 and 1. *T* is the maximum number of iterations of MOGA-LS. *t* is the current number of iterations of MOGA-LS. *ε* is a system parameter which is used to determine dependency degree on the number of iterations.

It is obvious that Δ*t* = (*U*_max⁡_^*k*^ − *X*_*k*_)(1 − *γ*_*k*_^(1−(*t*/*T*))^*ε*^^) ranges within [0, (*U*_max⁡_^*k*^ − *X*_*k*_)). To begin with, *t* is smaller, and thus, Δ*t* is larger, making the gene value have an obvious mutation. With the increase of the number *t* of iterations, the value of Δ*t* becomes gradually smaller. The change of the gene value becomes smaller. This feature makes the operator have ability in searching the whole space evenly in the early iterations (when *t* is small) and precisely searching several partial areas in the later iterations. To say further, the mutation operator makes the MOGA-LS approach have better global search ability in an early phase and have better local search ability and convergence in a later phase as it is a dynamic and self-adaptive mutation operator which can be adjusted by modifying the parameters such as *γ*_*k*_ and *ε*.

#### 3.2.5. Design of Fitness Values in MOGA-LS

(*1) The Fitness Value r*_*p*_. The design of the fitness values is the core of GA. And especially for multiobjective GA, it is more important since this kind of problems has multiple objective functions. In this paper, the Pareto dominance is utilized to achieve the Pareto ranking approaches and thus to obtain the fitness values of each individual in each generation. 

As mentioned above, the MOPs are the Pareto optimization problems, which have employed the Pareto dominance to compare and evaluate the individuals. The population is ranked according to the Pareto dominance rule, and then each solution is assigned a fitness value based on its rank in the population, not any one of its actual objective function values. Note that herein a lower rank corresponds to a better solution. The rank of each individual refers to its nondominated rank and is called *r*_*p*_. Each individual has a parameter *n*_*p*_, whichis the number of individuals that dominate the individual *p*. Each individual can be compared with every other individual in the population to find if it is dominated.

The rank *r*_*p*_ of each of the individuals whose *n*_*p*_ values are 0 is set as 1. At this stage, all individuals in the first nondominated front are found. In order to find the individuals in the next nondominated front, the solutions of the first front are discounted temporarily and the above procedure is repeated. The rank *r*_*p*_ of each of the individuals whose *n*_*p*_ values are 0 is set as 2, and so forth, until all the individuals of the population are ranked in this generation. After the nondominated sorting, the population is divided into several ranks and each individual has a rank *r*_*p*_. In other words, each of these ranks is a set consisting of several individuals. 

(*2) The Fitness Value D*_*p*_. The individuals between these ranks are comparable. In the same rank, the individuals cannot be compared with each other and are equal for being selected. Furthermore, we know that maintaining a diverse population is an important consideration in multiobjective GA to obtain solutions uniformly distributed over the Pareto optimal solutions set. Without taking preventive measures, the population tends to form relatively few clusters in multiobjective GA. This phenomenon which will prevent the MOGA-LS approach convergent to Pareto optimal solutions set is called genetic drift, in genetics. To further address the two problems of the fitness values and the diversity of a population, we have designed and employed the density estimation approach aiming to obtain a uniform spread of solutions along the Pareto front. Its main idea is that in the same rank the individuals in which there are fewer individuals around are better than others. It should be selected more potentially to generate the next generation in order to prevent genetic drift and keep the diversity of the population and thus to optimize the MOGA-LS approach. 

**Figure 1:**
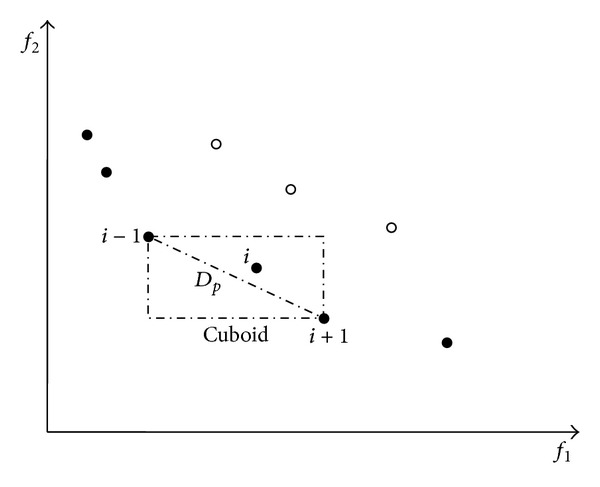
Crowding-distance calculation of the density estimation.

**Figure 2:**
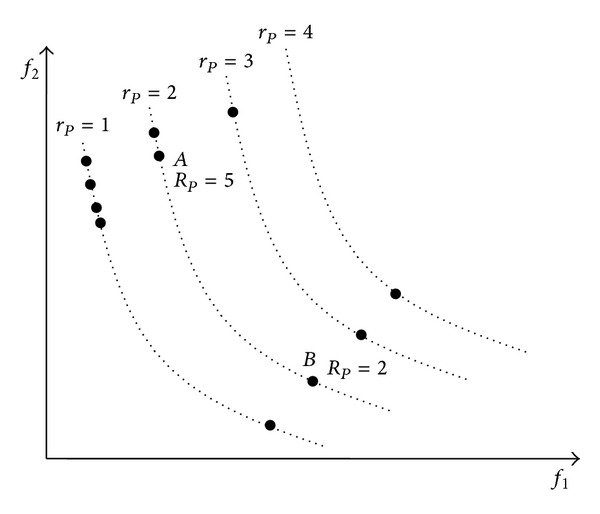
Individual rank values.

The process of the density estimation approach is as follows. To get an estimate of the density of solutions surrounding a particular solution in the population, we need to calculate the distance of two points on either side of this point along each of the objectives. This quantity *D*_*p*_ serves as an estimate of the diagonal of the cuboid formed by using the nearest neighbors as the vertices (call this the crowding distance). In this paper, we have defined the crowding distance of the *i*th solution in its front as the diagonal length of the cuboid as shown in Figure 1. The computation of the crowding distance needs to sort the population according to each objective function value in ascending order. Thereafter, for each objective function, the boundary solutions (solutions with smallest and largest function values) are assigned an infinite distance value. All other intermediate solutions are assigned a distance value equal to the corresponding diagonal length. If a MOP has three objective functions, the crowed distance is the diagonal length of a cube. If a MOP has two objective functions, the crowed distance is the diagonal length of a rectangle, and so forth. Each objective function is normalized before calculating the crowding distance.

(*3) The Fitness Value R*_*p*_. Now, each individual in the population has two attributes *r*_*p*_ and *D*_*p*_ in each generation. The Pareto rank *r*_*p*_ is the core of the MOGA-LS approach. Its convergence process is the process of converging the nondominated set of each generation to the Pareto optimal solutions set of the final generation. A rank represents a set of individuals where each element belongs to the nondominated rank. In other words, the individuals in the same rank are equal and have the same chances to be selected for generating the next generation. However, as a matter of fact, the individuals in the same rank are not equal due to their different situations of being dominated, especially for the individuals located in the ranks larger than 1. For each individual in the same rank, their *r*_*p*_ values are naturally equal. However, the *r*_*p*_ value does not reflect the ranks of the individuals dominating it. It is obvious that if the sum of the rank values of the individuals that dominate an individual is larger than that of other individuals in the same rank, the individual is not better for being used to generate the next generation. This is because its gene is not excellent enough and it is not beneficial for keeping the diversity of the population. Thus, the information included in the *r*_*p*_ value is not enough. We have presented a novel parameter *R*_*p*_. It is defined as follows:

(13) Rp=1+∑j=1tγXj,

where we assume that the individual *p* is dominated by *t* individuals *X*1, *X*2, …, *Xt*, whose rank values are already known as *γ*_*X*1_, *γ*_*X*2_, …, *γ*_*Xt*_. The dominated individuals are penalized to reduce the population density and redundancy. Here, we have given an instance. The multiobjective GA generates 11 individuals. Their rank values are illustrated in Figure 2. Suppose we want to minimize two objectives *f*_1_ and *f*_2_. Both *A* and *B* belong to rank 2. However, the *R*_*p*_ value of individual *A* is 5, and the *R*_*p*_ value of individual *B* is 2. The individual *B* is better. What is more, for the individuals that have the same ranks or the different ranks, *R*_*p*_ is more suitable as a fitness value for comparison since it has included not only the information of the nondominated rank of the individual but also the information of it being dominated for penalizing it. Meanwhile, it can be seen that the *R*_*p*_ value has also better kept the diversity of the population. From this perspective, it also makes up for the shortcoming of the density estimation since the density estimation approach is limited to the same rank. In order to achieve the calculation of *R*_*p*_, each individual *i* has a parameter *S*_*p*_, which is a set consisting of the *r*_*p*_ values of the individuals dominating the individual *i*.

Thus far, in our MOGA-LS approach, each individual of the population has 3 attributes *R*_*p*_, *r*_*p*_, and *D*_*p*_ as its fitness values in each generation. It can be seen that our design is that when comparing two individuals, the algorithm compares first their *R*_*p*_ values, second their *r*_*p*_ values, and last their *D*_*p*_ values. We now define a partial order ≺_*R*_*p*_,*γ*_*p*_,*D*_*p*__ as follows:

(14) i≺Rp,γp,Dp j if  (iRp<jRp)or  ((iRp=jRp),(iγp<jγp))or  ((iRp=jRp),(iγp=jγp),(iDp>jDp)).

The design not only has utilized the Pareto theory to achieve multiobjective comparison but also kept the diversity of the population, avoided genetic drift, and made the population more efficiently converged to the Pareto optimal solutions set with no additional complexity.

#### 3.2.6. System Architecture of MOGA-LS

**Figure 3:**
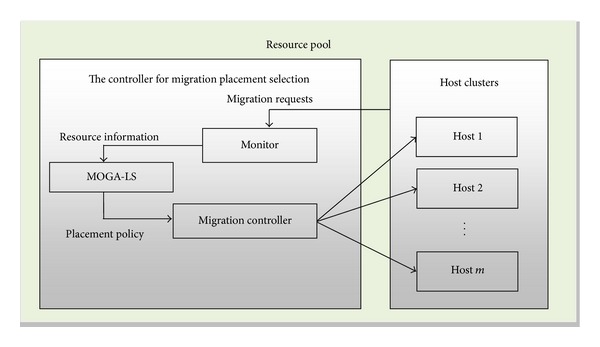
The view of MOGA-LS's architecture.

Figure 3 depicts the proposed architecture for cloud environment. It shows the position of the controller MOGA-LS for migration location selections and its interaction with other entities [13, 14]. In a time window Δ*t*, the monitor gets the requests of live VM migration and updated with the available number of computing resource such as CPUs, memory, and storage, as well as power consumption. At the end of Δ*t*, the monitor transfers the information to the controller. The controller generates the location policy by using the proposed approach and obtained information. Then, it transfers the policy to the migration controller which controls and executes live migration of the VMs. The VMs are eventually moved onto their target hosts.

### 3.3. Implementation of MOGA-LS

In this section, we describe the specific process of MOGA-LS. Details are as follows.

*Initialize the MOGA-LS Algorithm*. The size of the population is *N*. The number of the genes of an individual (a chromosome) is *n* due to the *n* migrant VMs as mentioned above. The maximum number of iterations (evolution) is *i*_max⁡_. The mutation rate of the population is *M*_*r*_. The competition scale of the tournament selection operator is set to 2. The initial population *P*_0_ is generated by employing the way in which the initial *archive* is generated in [15]. 

*Evolve the Population of MOGA-LS.* Each individual of *P*_0_ can be compared with every other solution to find if it is dominated and thus to obtain the *n*_*p*_ value of each individual by using the constrained fitness functions group (10). The algorithm sets the *γ*_*p*_ values of the individuals whose *n*_*p*_ values are 0 as 1. At this stage, all individuals in the first nondominated front are found. In order to find the individuals in the next nondominated front, the solutions of the first front are discounted temporarily and the above procedure is repeated. The rank *r*_*p*_ of each of the remaining individuals whose *n*_*p*_ values are 0 is set as 2, and so forth, until all the individuals of the population are ranked in this generation. 

After the nondominated sorting, the population is divided into several ranks and each individual has a rank value *r*_*p*_. Thereafter, each individual of *P*_0_ obtains its *R*_*p*_ value by using (13) and its *S*_*p*_ set. The algorithm first sorts the population *P*_0_ according to the first objective function (4) and the second objective function (9) in ascending order, respectively. The boundary solutions with smallest and largest function values are assigned an infinite *D*_*p*_ value. All other intermediate solutions are assigned a distance value *D*_*p*_ as follows:

(15) iDp =((i+1)·f1−(i−1)·f1f1max⁡−f1min⁡)2+((i+1)·f2−(i−1)·f2f2max⁡−f2min⁡)2.

Now each individual of the initial population *P*_0_ has the fitness values *R*_*p*_, *γ*_*p*_, and *D*_*p*_. The selection operator of MOGA-LS begins to perform the tournament selection of *P*_0_. Two groups of individuals are chosen randomly. Every group consists of two individuals. In each group, the individual with better fitness values is picked out according to the proposed partial order *r* : ≺_*R*_*p*_,*γ*_*p*_,*D*_*p*__. The crossover operator of MOGA-LS begins to perform the arithmetic crossover operation for the two winning individuals of the two groups according to (11) and thus to obtain two new individuals. The information of the Pareto nondominated rank values of the individuals of the population is introduced into the arithmetic crossover operator used by us. In the arithmetic crossover formula (11), the parameter *α* is defined as follows:

(16) $$\begin{array}{c} \alpha =\frac{j_{\gamma_{p}}}{i_{\gamma_{p}}+j_{\gamma_{p}}}. \end{array}$$

The crossover operator coefficient *α* is associated with the nondominated rank of each individual in the population. In the early iterations of MOGA-LS, the *α* value will have a great change. However, with the evolution of the population, the individuals of the population will tend to the same Pareto front and the *α* value will tend to the constant 0.5.

Repeat the selection and crossover operations until generating a population of size *N*. The mutation operator of MOGA-LS performs the dynamic nonuniform mutation for the generated population according to the mutation rate and (12) to eventually obtain the offspring *Q*_0_ of *P*_0_. At this time, the MOGA-LS algorithm forms a combined population *R*_0_ = *P*_0_ ∪ *Q*_0_. The size of the population *R*_0_ is 2*N*. Then, the population *R*_0_ is sorted according to nondomination rank. The elitism is ensured as all previous and current population members are included in *R*_0_. 

Now, the individuals belonging to the best nondominated set *S*_1_ are the best solutions in the combined population and must be emphasized more than any other solution in the combined population. If the size of *S*_1_ is larger than *N*, we utilized the *k*-means approach to cluster these individuals of *S*_1_ for grouping the solutions into *N* clusters. The *N* clustering centers of the *N* clusters are chosen to form the next population *P*_1_. If the size of *S*_1_ is smaller than *N*, we definitely choose all members of the set for the new population *P*_1_. The remaining members of the population *P*_1_ are chosen from subsequent nondominated fronts in the order of their ranking. Thus, solutions from the set *S*_2_ are chosen next, followed by solutions from the set *S*_3_, and so forth. This procedure is continued until no more sets can be accommodated. Assume that the set *S*_*l*_ is the last nondominated set beyond which no other set can be accommodated. In general, the count of solutions in all sets from *S*_1_ to *S*_*l*_ would be larger than the population size. To choose exactly *N* population members, we sort the solutions of the *last *front *S*_*l*_ according to the crowded distance *D*_*p*_ of the density estimation in descending order and choose the best solutions needed to make all population seats occupied. 

**Figure 4:**
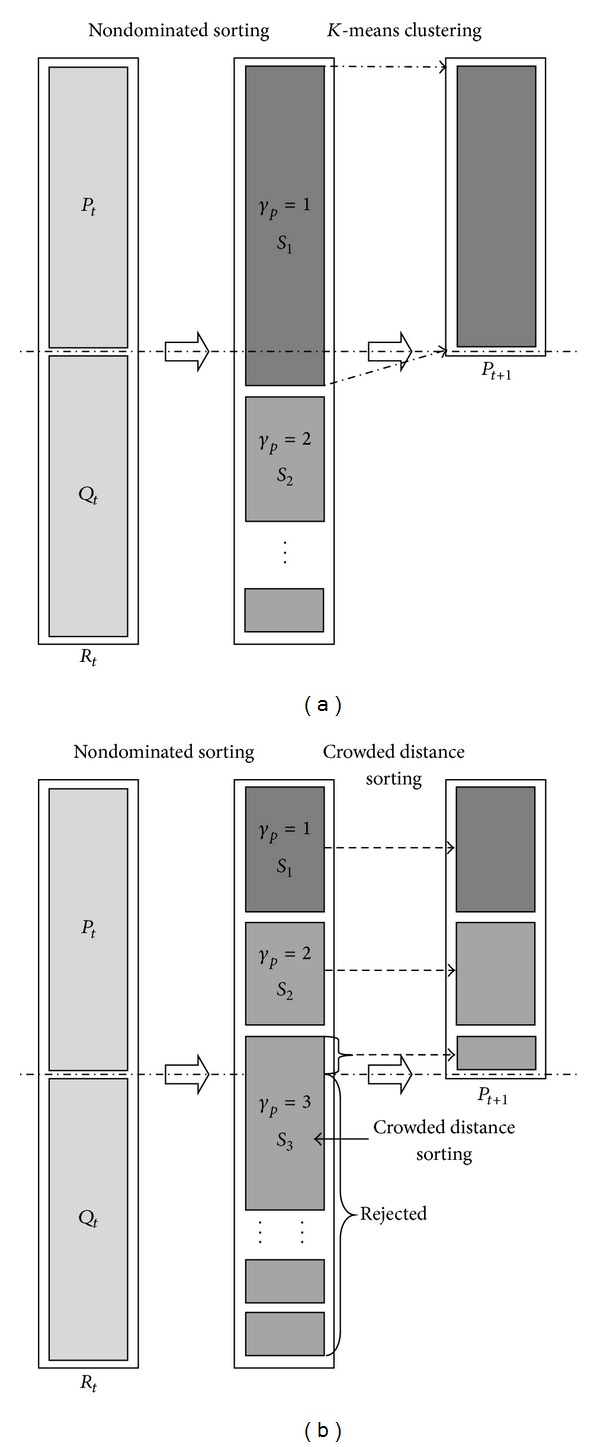
The procedure of generating the new population in MOGA-LS.

The procedure of the policy which is used for forming the next population and ensures elitism of population evolution in MOGA-LS is shown in Figure 4. The new population *P*_1_ of size *N* is now used for selection, crossover, and mutation to create a new population *Q*_1_ of size *N*. Repeat the above process until meeting the maximum number *i*_max⁡_ of iterations. That is, the maximum generation number *i*_max⁡_ of population evolution is reached. At that point, the final population obtained by evolving should be *P*_*i*_max⁡_−1_.

*Get the Final Solution Vector*. After the last round of iterations in MOGA-LS, the final population *P*_*i*_max⁡_−1_ is formed. The population *P*_*i*_max⁡_−1_ is sorted according to nondomination rank. Its first nondominated set (*γ*_*p*_ = 1) is the final Pareto optimal solutions set. Although the set of solutions vectors is the final solutions set of the multiobjective optimization problem proposed by us on the location selection policy of live VM migration, the migrant VMs cannot be migrated according to multiple solutions.

Generally, the way to address this problem is to choose a solution randomly or according to the context from the Pareto optimal set. However, as for the proposed Pareto multiobjective optimization problem of live VM migration policy in cloud environment, MOGA-LS is a heuristic approach which does not have and does not need any context information, and it should be a self-adaptive algorithm. Besides, randomly choosing a solution from the Pareto optimal set is nonefficient and one-sided.

In this paper, we have presented a novel approach to address the problem of selecting a solution from the obtained set of Pareto optimal solutions. Now, assume that there are *L* solutions vectors in the Pareto optimal set. The *L* solutions vectors can constitute a matrix Θ of *L*∗*n*, where each row vector is a solution vector belonging to the Pareto optimal set. Each of the *n* column vectors of the matrix Θ represents *L* solutions of the location selection of the corresponding VM. For each of the *n* migrant VMs, it has a solution space consisting of *L* solutions. The *L* solutions should consist of a few kinds of solutions. The probability of each solution is obtained by calculating their frequency of occurrence in the *L* solutions according to probability theory and mathematical statistics. 

**Figure 5:**
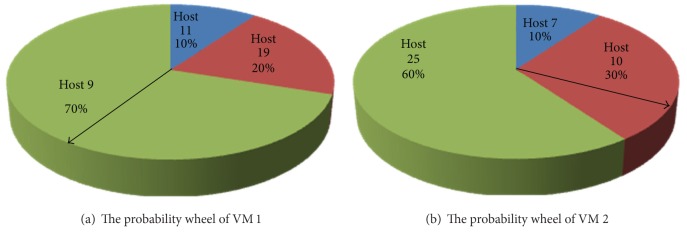
Examples of the probability wheel.

At this point, each migrant VM has a probability wheel as shown in Figure 5. A pointer randomly rotates on the probability wheel. The host in which the area where the pointer finally stops represents is the final target host of live migration. In the specific implementation, a random number limited between 0 and 1 can be used to find the final location of live VM migration by finding the range where the random number is. By utilizing this approach, each migrant VM gets the final solution of location selection of live VM migration. An *n* dimension of solution vector is returned to migration controller as an optimal migration policy of the *n* migrant VMs.

Let us consider a problem in the proposed approach. We know that the solution vector of the proposed problem ought to be a sequence of integer numbers which denote the hosts in the resource pool. However, each individual of the initial population is randomly initialized. What is more is that the crossover and mutation functions have some coefficients which are limited between 0 and 1. Thus, although the genes of each individual are limited to an integer type in the implementation, the problem that the solution is not an integer still persists. To address this problem, we do not limit the elements to an integer type but employ the smallest position value (SPV) rules presented by in [16, 17]. In short, it is a rule which converts each individual given by the GA to a valid solution vector fit for the proposed problem. The process of applying the SPV rules into the proposed approach can be understood as follows. First, the hosts in the resource pool should be numbered from 0 to *m* − 1. Second, after the initial population is initialized, all the genes of each individual are sorted in ascending order and then are numbered from 0 to *n* − 1 and have a modulo operation of *m*. For instance, in a time window Δ*t*, there are four VMs to be migrated in a resource pool which has three hosts. If an individual is (−1.21, 3.29, −0.12, 1.26), it will be converted to (0, 3, 1, 2) firstly and then to (0, 0, 1, 2). At this point, the solution vector is useful and meaningful. It represents that the target host of VM 0 is host 0. The target host of VM 1 is host 0. The target host of VM 2 is host 1. The target host of VM 3 is host 2. In the MOGA-LS, all the solutions vectors (individuals) refer to the vectors which the original vectors have been converted to according to the SPV rules.

Starting from this intuition, the MOGA-LS algorithm which aims at live VM migration policy gives consideration to both the power saving optimization and the load balancing optimization to achieve a more efficient cloud data center by designing and utilizing the GA. During the course of the study, we have found that the two goals compete with each other to further improve itself. Thus, the GA cannot be utilized directly into our proposed problem. At this point, based on some inherent contradictory relationship between the two goals, we have thought of game theory, which is aiming to deal with and research this kind of problems. In the process that we further study game theory and multiobjective optimization, we have found the Pareto dominance theory. Based on the research on the Pareto dominance theory, we have proposed a novel multiobjective GA fit for being used to address the proposed problem and designed its genetic operators and fitness values as well as the policy of generating the next population. The specific process of MOGA-LS is also presented. In the MOGA-LS algorithm, each subalgorithm and subprocess are specially designed to better achieve our proposed two optimization goals. Therefore, MOGA is an obviously efficient heuristic algorithm for location selection policy of live VM migration from the experimental and design's points of view.

In the proposed MOGA-LS approach, we have introduced the simulated annealing (SA) idea [18] into the process of selecting the final solution from the obtained Pareto optimal set. Since the MOGA-LS algorithm is based on the Pareto dominance theory, the returned solution of MOGA-LS should be a set of Pareto optimal solutions (Pareto optimal front). In our problem scenario, it is very natural that only one final solution should be obtained as each VM needs to finally identify which host it will be migrated onto clearly. In the set of Pareto optimal solutions, it is obvious that all the solutions are equal and not comparable. Thus, the idea of comparing these solutions through other respects is meaningless and putting the cart before the horse. If so, we may as well directly add a new goal in the proposed MOP. Finally, the problem still persists. Thus, we think the way that a final solution is selected by comparing all the solutions of the Pareto optimal front does not work. The widely employed way is to randomly selecting a solution from the Pareto optimal front. Its rationality lies in that all the Pareto optimal solutions are optimal, equal, and not comparable as mentioned above. Although the method is simple and addresses this problem in a way, randomly choosing is only an expedient and obviously not efficient. Furthermore, our problem scenario is also not related to any context information to help MOGA-LS select the final solution, and the MOGA-LS approach is a self-adaptive heuristic algorithm, which needs to achieve the selection of the final solution autonomously. To address this problem in MOGA-LS, we have utilized the SA idea to convert the seeming disadvantage that multiple optimal solutions (Pareto optimal solutions set) are returned at last to the advantage that each VM finally obtains a sample space of optimal solutions and can be migrated to some optimal target host with a certain probability and thus to achieve a long-term optimization by implementing the SA idea. The specific process is shown above. 

There are many parameters in the proposed MOGA-LS algorithm. Most of them have great influences on MOGA-LS. The maximum number *i*_max⁡_ of iterations of the population evolution and the population size *N* of MOGA-LS are two important parameters as well as their values are related to the efficiency and accuracy of MOGA-LS. For instance, if the population size *N* increases, the computation time (convergence time) of MOGA-LS will increase, and the possibility that the algorithm is converged to the Pareto optimal front will also increase. That is, the global search ability will strengthen. Moreover, the required generation number *i*_max⁡_ of population evolution will decrease with the population size *N* increase. The maximum number *i*_max⁡_ of iterations of the population evolution should be neither too small nor too large. Evidently, the size of the *i*_max⁡_ value should make the first Pareto nondominated solutions set of the population of the MOGA-LS algorithm exactly converged to the Pareto optimal front as much as possible. Therefore, both the *i*_max⁡_ value and the *N* value are the experience problems and large numbers of experiments need to be performed to obtain fit values of them. In the proposed MOGA-LS approach, *N* is set to 20 and *i*_max⁡_ is set to 100 based on a large number of experiments. Also, the mutation rate *M*_*r*_ is another important parameter in MOGA-LS since it has a direct impact on the diversity and convergence of population in MOGA-LS. It is difficult for GA to assign the mutation rate *M*_*r*_ a fit value as it is related to a number of factors. Thus, it is an open problem which needs be studied further. In this paper, the *M*_*r*_ value is set to 0.4. That is, there are 8 individuals to be mutated in each round of iterations in our proposed MOGA-LS approach.

## 4. Evaluation

In this section, we have experimentally verified the proposed MOGA-LS approach. The experiments have included evaluating the effect of power saving and load balancing of MOGA-LS and verifying the performance of MOGA-LS migration policy on the failure rate of migration events and SLA violation. Besides, an auxiliary experiment has been conducted to get a best power management policy in the cloud data center implementing the proposed MOGA-LS policy. In order to simulate a dynamic cloud data center, we have utilized an event driven simulator named CloudSim toolkit [19]. The CloudSim framework enables the accounting of the total power consumed by the system during the simulation period. In one word, the calculating of power consumption has been achieved by the CloudSim platform, which has provided a class including the methods getPower(). CloudSim allows such simulation scenarios by supporting dynamic creation of different kinds of entities and can add and remove data center entities at run time. This functionality has achieved simulating dynamic cloud environment where system components can join, fail, or leave the system randomly [19]. On CloudSim platform, we compare the proposed MOGA-LS approach with random migration policy and optimal migration policy by power consumption, the degree of load balancing and the number of invalid VM migration. We have prepared four different kinds of experiments to evaluate and test the proposed approach. The experimental results demonstrate that the proposed approach not only has a better power saving and load balancing but also has a better migration performance. Especially for a large number of migration events, the MOGA-LS approach shows the stability and the better performance.

### 4.1. Experimental Scenarios

On CloudSim platform, a resource pool consisting of 100 hosts is created. These hosts have varying computing resource. Twenty-four batches of virtual machine migration requests containing 13 requests randomly belonging to different hosts and with different resource requirements are created. The proposed MOGA-LS module is invoked and fetches the resource information and state of the cloud resource pool periodically. Δ*t* is set as 600 seconds, and the migration events of each batch are uniformly distributed within an hour, which includes 6Δ*t* s. Besides, the host's resource change rate is set as 1 time per half an hour.

### 4.2. Comparison in Power Saving

**Figure 6:**
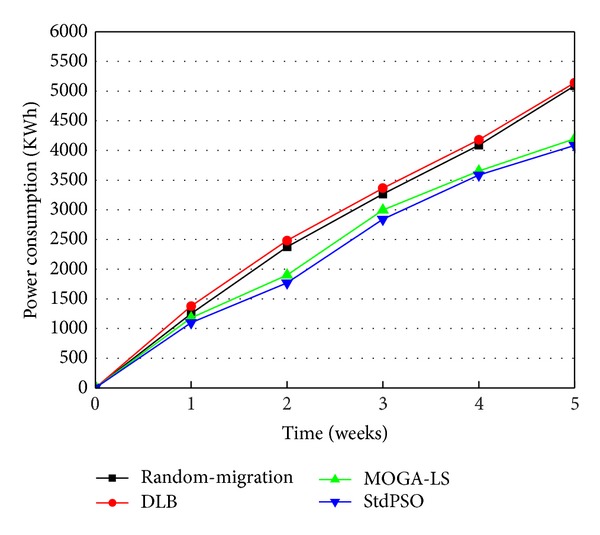
Comparison of random-migration, DLB, StdPSO, and MOGA-LS in power consumption.

The experiment is designed for verifying the efficiency and availability of MOGA-LS in power saving due to the location selection of live VM migration during the long-term operation of a cloud data center. In this scenario, we compare MOGA-LS with random migration policy, dynamic load balancing (DLB) policy implementing the idea of load balancing in [20], and the StdPSO migration policy which is an optimal migration policy based on standard particle swarm optimization for only power saving by power consumption during the long-term operation of the simulated cloud data center. As illustrated in Figure 6, the cloud data center implementing DLB migration policy and the cloud data center implementing random migration policy consume more power, while the cloud data center implementing MOGA-LS and the cloud data center implementing the StdPSO migration policies consume relatively less power. This is because both the latter two are the heuristic approaches for power saving. Further, the cloud data center implementing DLB migration policy causes more power consumption than that of random migration policy. The reason for this is that the DLB migration policy has only taken load balancing into account. Achieving the balancing of load is to improve the service performance of cloud data center. What it has been considering is the user side. The migration policy for only the above goal naturally causes relatively more power consumption than the random migration policy since the two objectives, power consumption, and load balancing are competing with each other, and the random migration policy does not sway the balance on either side. 

Besides, as can be seen in Figure 6, the cloud data center implementing the StdPSO migration policy has a less total incremental power consumption than the cloud data center implementing the MOGA-LS migration policy. The MOGA-LS approach has considered not only power saving but also load balancing. Just from the point of view on power-saving effect, the MOGA-LS policy is not better than the StdPSO policy as mentioned above. However, the power-saving effect of the MOGA-LS policy is very close to that of the StdPSO policy in Figure 6. The MOGA-LS algorithm has achieved many proposed optimization policy and thus had a better convergence to the Pareto optimal front and has utilized the SA idea to achieve a long-term optimization effect, effectively avoiding the local optimization. Therefore, although it has also considered load balancing, it obviously still has a better power-saving effect than the random migration policy and the DLB policy. Moreover, its power saving effect approximates that of the StdPSO algorithm for optimally saving power. To sum up, the proposed MOGA-LS approach is an efficient migration policy of live VM migration for power saving.

### 4.3. Comparison in Load Balancing

**Figure 7:**
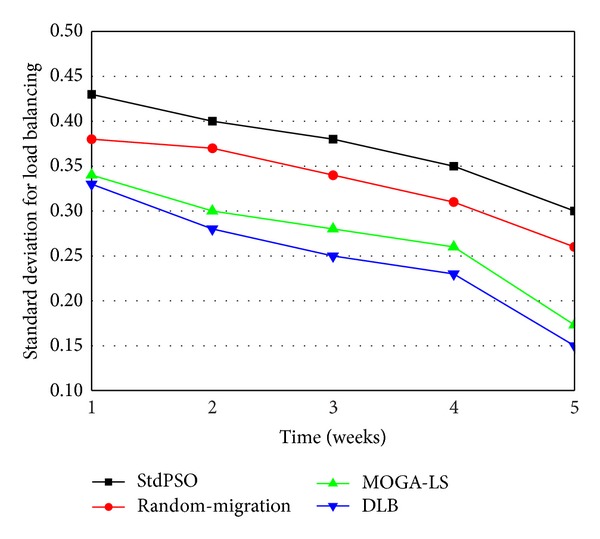
Comparison of StdPSO, random-migration, DLB, and MOGA-LS in load balancing.

In the experiment scenario, it is designed for verifying the efficiency and availability of MOGA-LS in load balancing due to the location selection of live VM migration during the long-term operation of a cloud data center. We have compared the degrees of load balancing in the cloud data centers implementing the StdPSO policy, random migration policy, MOGA-LS migration policy, and DLB migration policy, respectively, at the end of each of five weeks. Here, we have employed the standard deviation value indicated above and used to measure the degree of load balancing to conduct the experiment. Obviously, a smaller standard deviation value represents that the cloud data center has the better balancing of load. We know that in a cloud data center there is the dedicated load balancing algorithm running regularly in order to actively achieve the balancing of system load. Thus, the degree of load balancing of a cloud data center will be getting better and better to some extent with the time passing. As shown in Figure 7, all the four policies' standard deviation values measuring the balancing of load have been tending to become smaller to some extent. However, at the end of each week, the obtained four policies' standard deviation values have shown that there are the certain gaps between the degrees of load balancing of cloud data centers. In Figure 7, the cloud data center implementing the StdPSO policy has the worst degree of load balancing. The reason is similar to the previous experiment. The cloud data center implementing the random migration policy has the second worst effect of load balancing, relatively, since it does not have any information. The DLB migration policy leads to the best effect of load balancing relatively. Meanwhile, the MOGA-LS approach is close to it and has the second best effect of load balancing. As can be seen in Figure 7, the four migration policies are divided into two levels of load balancing. In other words, the MOGA-LS migration policy and the DLB migration policy are the same level although the MOGA-LS algorithm is not as excellent as the DLB algorithm. It is obvious that the MOGA-LS approach has already been quite efficient for load balancing. To sum up, the proposed MOGA-LS approach is an efficient and feasible migration policy of live VM migration for load balancing.

### 4.4. Comparison of the Number of Failures in VM Migration Events

**Figure 8:**
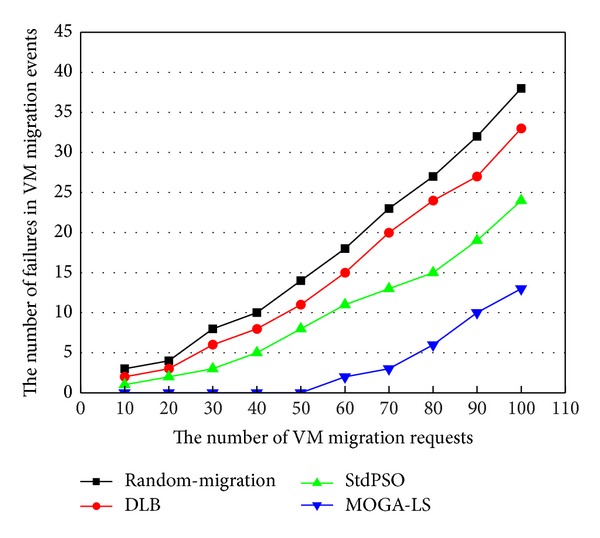
Comparison of the number of failures in VM migration events.

In the experiment scenario, the dynamic host failure is simulated by CloudSim by scheduling some host failure events and host shut down events to occur during the location selection interval. These events have caused some failures in VM migration due to the nonavailability of the selected hosts for some VM migration requests. As illustrated in Figure 8, we have compared the MOGA-LS policy with the random migration policy, the DLB migration policy, and the mentioned StdPSO migration policy. In the simulated cloud data center, with the increase of the number of VM migration requests, the cloud data centers, respectively, implementing random migration, DLB and StdPSO result in more number of invalid VM migrations, whereas the MOGA-LS performs better in finding the fit hosts in the dynamic resource pool. This is because the memory data is outdated in the StdPSO, DLB, and random migration policy. They cannot have an adjustment with the environment changed. Conversely, since the SA idea has been introduced into it, the MOGA-LS policy based on the GA is quite efficient in detecting the host failures during the interval as well as has a fit adjustment in a better manner by searching out the new available hosts that can meet the resource requirements of the VM migration requests. The MOGA-LS approach can better meet the performance requirement of live VM migration and reduces the failure numbers of live VM migration since the MOGA-LS algorithm has seen the migration performance as the constraint objective. The design of the MOGA-LS approach is rational and efficient. After all, it is the migration policy of VMs that we are discussing. And it should first make live migration of VMs succeed as much as possible.

### 4.5. Comparison of the Incremental Cost due to SLA Violation

**Figure 9:**
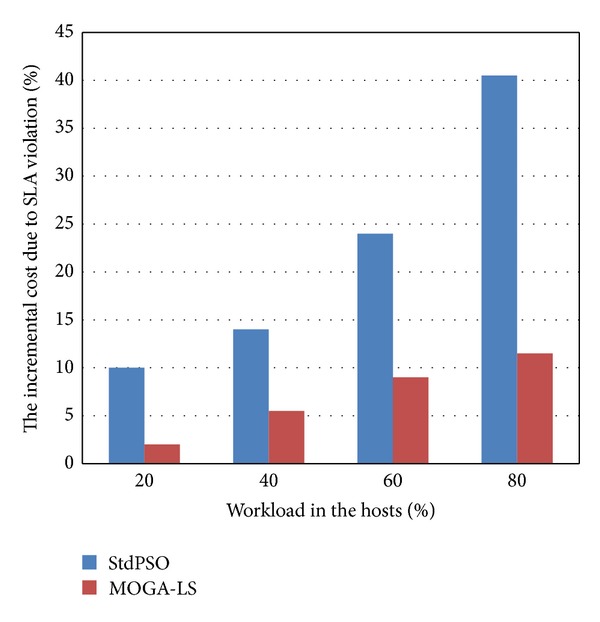
Comparison of the incremental cost due to SLA violation in VM migration with varying percentage of load in the cloud data center.

In this experimental scenario, we compare the incremental cost of StdPSO and MOGA-LS due to service level agreements (SLA) violation with varying percentage of load in the cloud data center. The load mentioned refers to the load of the whole cloud data center and is not the load of some physical host. As illustrated in Figure 9, with the increase of the percentage of workload in the cloud data center, the incremental cost of MOGA-LS is less than that of StdPSO due to SLA violation. We know if the workload in the hosts is heavier, achieving the balancing of load is more important for better service performance and to, thus, meet the SLA. The above experiment has shown that the StdPSO policy and the MOGA-LS policy belong to the same level for power saving. MOGA-LS is just slightly inferior than StdPSO. However, since the proposed MOGA-LS approach has also achieved the balancing of load, it has ability in making the cloud data centers increase resource utilization and provide better service performance to users. Thus, its cost due to SLA violation is less. So to say, the MOGA-LS approach has not only made a quite excellent power-saving effect come true to contribute to the green cloud data centers but also is considered the most important user experience and achieved load balancing and thus to enhance the resource utilization and service capability of green cloud data centers. To sum up, the MOGA-LS algorithm is an efficient location selection policy of live VM migration. 

### 4.6. Tradeoff between Power Saving and Performance Fulfillment

**Figure 10:**
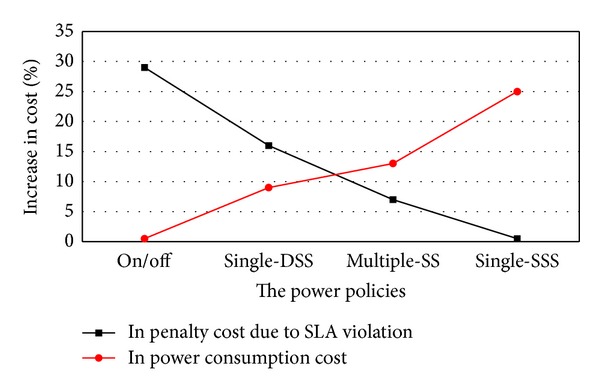
The trade-off between power and performance with different power management policies.

The experimental scenario is conducted to find the optimal power management policy which balances the benefits due to power saving and performance fulfillment. Since live VM migration events are time critical VM requests and the cloud service provider should meet strict SLA compliance, any violations in SLA in terms of performance loss of VMs will result in penalty cost on the provider of a cloud data center. The performance loss mentioned may be caused by power saving, live VM migration, network bandwidth, and throughput, and so forth. However, this paper has not focused on these problems. This experiment is designed to only find out a better power management policy suitable for the cloud environment implementing the proposed MOGA-LS to have a trade-off between the benefit of power saving and penalty cost due to SLA violation. In other words, the experiment aims to find a power management policy, which makes the proposed MOGA-LS approach more efficient in the cloud data centers and makes the cloud data centers implementing the MOGA-LS migration policy have a better power-saving effect and satisfy the users. There are four different policies to be formulated. The first policy is on/off policy, wherein all idle hosts are switched off. It can be seen that the policy gives the best power saving, but it causes the high penalty cost obviously. The single-DSS policy is the second policy, wherein all idle hosts are switched to deep sleep state. It results in an increase in the power consumption cost, but the penalty cost is reduced significantly. The third policy is single-SSS, wherein all idle hosts are switched to shallow sleep state. There is no penalty cost as SLA violation is absent in this policy. However, the enormous increase in the power consumption cost is caused. The multiple-SS is the fourth policy, wherein some of the idle hosts are kept in deep sleep, and others are kept in shadow sleep state according to a short-term prediction technology. From its meaning and as shown in Figure 10, it can be known that the multiple-SS policy gives the optimal cost trade-off, relatively.

## 5. Conclusion and Future Work

In this paper, a novel location selection policy MOGA-LS of live VM migration is proposed, and we give its main idea, design, implementation, and evaluation. It employs the improved GA-based approach and the Pareto dominance idea. In the improved GA-based approach, we have designed the selection crossover, the crossover operator, and the mutation operator of MOGA-LS. Moreover, we have designed the fitness values of MOGA-LS and given how to obtain them. Also, in order to make the MOGA-LS approach have the elitism, we have designed and employed a novel (*μ* + *λ*) selection policy based on Pareto nondominated sorting to generate the next population and thus to further optimization the MOGA-LS algorithm. 

It is noteworthy that in the proposed MOGA-LS approach we do not randomly get the initial population but employ a novel optimization method utilizing the hill-climbing technique and clustering technique. In this paper, we do not give its details. Besides, in the process of generating the next population, we have introduced *K*-means clustering into the process to address this problem that the number of the individuals of the first nondominated set may be larger than *N*. In the proposed MOGA-LS, we have also utilized the SA idea to obtain the final solution vector and achieve a long-term optimization. Also, aiming to connect the improved GA-based algorithm and the SA process, we take use of the probability theory and mathematical statistics as well as the characteristics (the returned solution is the set of Pareto optimal solutions) of the algorithm itself to obtain and process data.

MOGA-LS achieves the high-efficiency of power consumption and the stability of requirement performance. It not only minimizes the incremental power consumption of a cloud data center but also achieves the balancing of system load while minimizing the number of failure in VM migration events, relatively. In the proposed MOGA-LS approach, there are some open problems which need be studied further and experience problems which need many experiments to gradually get a better solution. The maximum number *i*_max⁡_ of iterations of the population evolution and the population size *N* of MOGA-LS are the experience problems and need to perform several experiments to obtain the fit values to, thus, make the MOGA-LS approach efficient and feasible. The mutation rate *M*_*r*_ of our approach is an open problem which needs be further widely researched. In this paper, all the parameters are set to the fit values, respectively. To evaluate the MOGA-LS approach, we have conducted several experiments on the CloudSim platform. The final experimental results show that MOGA-LS has an excellent power-saving effect and achieves the balancing of system load while having a high success rate of live VM migration events. Therefore, MOGA-LS is an effective, heuristic, and self-adaptive location selection policy of live VM migration for power conservation and load balancing.

Aiming to further improve the performance of MOGA-LS, we plan to study the robustness of MOGA-LS in the next step work. MOGA-LS should have abilities in dealing with some sudden matters and be combined with the mechanism of live VM migration for achieving a more efficient hybrid management. In the next work and experiments, the experience problems and the open problems presented in this paper will also be researched further.
